# Plasma bradykinin and early diabetic nephropathy lesions in type 1 diabetes mellitus

**DOI:** 10.1371/journal.pone.0180964

**Published:** 2017-07-10

**Authors:** Kevin M. Wheelock, Jian Cai, Helen C. Looker, Michael L. Merchant, Robert G. Nelson, Gudeta D. Fufaa, E. Jennifer Weil, Harold I. Feldman, Ramachandran S. Vasan, Paul L. Kimmel, Brad H. Rovin, Michael Mauer, Jon B. Klein, for the CKD Biomarkers Consortium

**Affiliations:** 1 National Institute of Diabetes and Digestive and Kidney Diseases, Phoenix, Arizona, United States of America; 2 University of Louisville, Louisville, Kentucky, United States of America; 3 University of Pennsylvania, Philadelphia, Pennsylvania, United States of America; 4 Boston University School of Medicine, Boston, United States of America; 5 National Institute of Diabetes and Digestive and Kidney Diseases, Bethesda, Maryland, United States of America; 6 Ohio State University, Columbus, Ohio, United States of America; 7 University of Minnesota, Minneapolis, Minnesota, United States of America; University of Glasgow, UNITED KINGDOM

## Abstract

**Objective:**

To examine the association of bradykinin and related peptides with the development of diabetic nephropathy lesions in 243 participants with type 1 diabetes (T1D) from the Renin-Angiotensin System Study who, at baseline, were normoalbuminuric, normotensive and had normal or increased glomerular filtration rate (GFR).

**Design:**

Plasma concentrations of bradykinin and related peptides were measured at baseline by quantitative mass spectrometry. All participants were randomly assigned at baseline to receive placebo, enalapril or losartan during the 5 years between kidney biopsies. Kidney morphometric data were available from kidney biopsies at baseline and after 5 years. Relationships of peptides with changes in morphometric variables were assessed using multiple linear regression after adjustment for age, sex, diabetes duration, HbA1c, mean arterial pressure, treatment assignment and, for longitudinal analyses, baseline structure.

**Results:**

Baseline median albumin excretion rate of study participants was 5.0 μg/min, and mean GFR was 128 mL/min/1.73 m^2^. After multivariable adjustment, higher plasma concentration of bradykinin (1–8) was associated with greater glomerular volume (partial r = 0.191, *P* = 0.019) and total filtration surface area (partial r = 0.211, *P* = 0.010), and higher bradykinin (1–7) and hyp3-bradykinin (1–7) were associated with lower cortical interstitial fractional volume (partial r = -0.189, *P* = 0.011; partial r = -0.164, *P* = 0.027 respectively). In longitudinal analyses, higher bradykinin was associated with preservation of surface density of the peripheral glomerular basement membrane (partial r = 0.162, *P* = 0.013), and for participants randomized to losartan, higher hyp3-bradykinin (1–8) was associated with more limited increase in cortical interstitial fractional volume (partial r = -0.291, *P* = 0.033).

**Conclusions:**

Higher plasma bradykinin and related peptide concentrations measured before clinical onset of diabetic nephropathy in persons with T1D were associated with preservation of glomerular structures, suggesting that elevations of these kinin concentrations may reflect adaptive responses to early renal structural changes in diabetic nephropathy.

## Introduction

Diabetes is the leading cause of chronic kidney disease and end-stage renal disease (ESRD) in the United States [1]. Reducing the burden of diabetic kidney disease requires early identification and treatment of persons with diabetes at increased risk for diabetic nephropathy (DN). Elevated urinary albumin excretion is considered the best marker of early progressive DN, but loss of renal function may precede the rise in albumin excretion [2] and elevated albumin excretion may only appear after significant structural damage has already occurred [3]. Given these uncertainties, other markers may improve risk prediction in individuals with diabetes.

Structural changes within the kidney precede and predict clinical DN [3,4] so biomarkers that predict these structural changes may be of great clinical value. We previously reported that elevated urinary concentration of monocyte chemoattractant protein-1, a marker of inflammation that predicts renal function decline and progression to ESRD in persons with diabetes [5], was associated with interstitial changes over 5 years in the kidneys of predominantly white women with type 1 diabetes (T1D) and no clinical evidence of DN [6]. We also found that elevated serum concentrations of tumor necrosis factor receptors 1 and 2, which are strong independent predictors of renal function decline leading to ESRD in persons with diabetes [7,8], are associated with early mesangial expansion and loss of glomerular capillary endothelial cell fenestrations in Pima Indians with type 2 diabetes [9]. Together, these studies support further discovery efforts to identify potential biomarkers of early structural damage in DN.

Bradykinin is believed to play an important role in protecting the kidney from damage caused by diabetes, but paradoxically it may also play a role in the development of DN. Increased concentrations of bradykinin precursors are associated with progressive renal dysfunction in persons with T1D [10] but the nature and timing of bradykinin’s renoprotective versus destructive roles are uncertain. The bradykinin 2 receptor (B2R) mediates most of the physiological actions of bradykinin, which are generally considered renoprotective [11, 12]. On the other hand, increased activation in diabetes of the bradykinin 1 receptor (B1R), is mainly associated with increased inflammation and worsening of kidney disease [12,13]. The bradykinin family of peptides may therefore be useful biomarkers for DN, perhaps initially through salutary adaptive responses to early DN, primarily mediated by B2R, and later through pathological responses to inflammation and fibrosis in more advanced disease, predominantly mediated by B1R.

Bradykinin has a half-life in the circulation of under 30 seconds [14], and is quickly metabolized to a number of related peptides which differ in their affinities for B1R and B2R. For example, bradykinin binds primarily to B2R, whereas its metabolite bradykinin (1–8) has stronger affinity for B1R than B2R, and bradykinin (1–7) and bradykinin (1–5) are inactive forms that do not activate either receptor. The bradykinin family of peptides also includes forms where the third proline is hydroxylated, and these hydroxylated forms have very similar activity profiles to their non-hydroxylated forms [15]. Angiotensin converting enzyme (ACE) is a key enzyme in the metabolism of bradykinin to inactive forms such as bradykinin (1–7) and bradykinin (1–5), and both the ACE inhibitors and angiotensin receptor blockers (ARBs) are associated with increased bradykinin concentrations [16,17].

The Renin-Angiotensin System Study (RASS) was a multicenter double blinded placebo controlled clinical trial designed to test whether renin angiotensin system blockade with either enalapril or losartan slowed progression of DN lesions over 5 years in normotensive, normoalbuminuric individuals with T1D and normal or increased glomerular filtration rate (GFR). Participants had a research kidney biopsy performed at enrollment and again at the end of the 5-year study, and the primary endpoint was a change in the fraction of glomerular volume occupied by mesangium [Vv(Mes/glom)]. Change in Vv(Mes/glom) over the 5-year period did not differ significantly between the placebo and the two treatment groups, and there were also no significant treatment benefits for the other structural variables that were assessed [18]. In the present study, we measured six peptides of the bradykinin family in stored plasma collected from RASS participants during the clinical trial––bradykinin (BK), BK(1–7), BK(1–8) and their respective hydroxylated forms hyp3-BK, hyp3-BK(1–7) and hyp3-BK(1–8), and we examined their association with kidney structural variables and the changes in these variables during the 5 years of RASS.

## Materials and methods

Of the 285 participants aged 16–65 years enrolled in RASS, 243 had both a baseline and follow-up kidney biopsy plus baseline plasma samples available for measurement of bradykinin and related peptides (S1 Fig). No participants were exposed to renin angiotensin blockade or to any other antihypertensive therapy prior to enrollment. After the baseline examination and kidney biopsy, participants were randomly assigned to receive either placebo, enalapril or losartan. Clinical measures available from the baseline examination included blood pressure, HbA1c, albumin excretion rate (AER) measured from timed overnight urine collections, and GFR measured directly by the plasma disappearance of iohexol (iGFR) [19]. Mean arterial pressure (MAP) was calculated as (2×diastolic blood pressure + systolic blood pressure)/3. The RASS study was conducted complying with the Declaration of Helsinki. Written informed consent was obtained from all participants. For participants under the age of 18 at study enrollment (lowest age was 16 years), written consent was obtained from the parents or legal guardians and written assent from the participants. The institutional review board at each study site approved the study.

### Morphometric measurements

Renal structural parameters of DN were measured using masked systematic unbiased random sampling morphometric methods [20,21]. Point counting on electron microscopic (EM) images was used to measure Vv(Mes/glom). Peripheral glomerular basement membrane surface density [Sv(PGBM/glom)], which reflects glomerular filtration surface area, was measured by an EM line intercept method, and GBM width was measured by the EM orthogonal intercept method. Point counting on periodic Schiff stained light microscopic (LM) slides of Zenker’s fixed material was used to measure the fraction of renal cortical volume occupied by the interstitium [Vv(Int/cortex)]. Measurement of Vv(Int/cortex) required substantial amounts of tissue, because valid estimates required at least 65 coarse grid points to fall on renal cortex [22]. Of the 243 participants with dual kidney biopsies and baseline measurement of bradykinin and related peptides, 189 had sufficient tissue for measurement of V_V_(Int/cortex) at both the baseline and 5-year biopsies. A further subset of 156 had a baseline measurement of glomerular volume (GlomV) using the Cavalieri method [23], although there were too few (n = 37) with a repeat measure at 5 years to allow for analysis of change in GlomV. Total filtration surface per glomerulus (TFS/glom) was calculated by multiplying Sv(PGBM/glom) by GlomV.

### Measurement of plasma bradykinin and related peptides

Baseline plasma specimens stored at -80°C were assayed in 2014 for bradykinin and related peptides by differential mass spectrometry (http://www.ckdbiomarkersconsortium.org/laboratory_SOPs.pdf). Assay reproducibility was assessed by intra-class correlation of measurements from 20 duplicate samples blinded to the performance laboratory. Intra-class correlations for bradykinin and related peptides ranged from 0.97–0.99, reflecting excellent agreement.

### Statistical analyses

Bradykinin and related peptides were log transformed to approximate normality prior to analysis (S2 Fig). All structural measures were log-transformed prior to analysis and changes in morphometric variables were computed as the logarithm of the 5-year structural measure minus the logarithm of the baseline measure (equal to the logarithm of the ratio of 5-year biopsy measurement to the baseline measurement).

Cross-sectional relationships between baseline bradykinin and related peptides and each morphometric variable were assessed by Pearson correlations and after adjustment for age, sex, duration of diabetes, HbA1c, MAP, and treatment assignment by multiple linear regression and partial Pearson correlations. For longitudinal relationships, the baseline value of the morphometric variable was also included in the linear regression model.

Concentrations of bradykinin and its related peptides were combined for three summary measures: sum of i) all peptides, ii) hydroxylated peptides and iii) unmodified peptides. These summary measures were analyzed to determine whether total plasma kinin abundance, regardless of its form, correlated with changes in morphometric variables. We tested for interactions with sex in all regression models and for interactions with treatment assignment in models of change in morphometric measures, as the use of enalapril or losartan during the study could in theory impact associations between baseline kinins and changes in morphometry. These interactions were deemed statistically significant if the interaction term included in the model had a p-value <0.05.

Two sensitivity analyses were performed. In one, we replaced the change in each morphometric variable with the 5-year measurement value. In the other, we added AER and iGFR to the linear regression models. These variables were not included in our primary models, since they correlate with the underlying structure.

## Results

Clinical and demographic characteristics of the participants, and the median and IQRs for the bradykinin peptides, are shown in Table 1. The range of values for each bradykinin peptide is shown in S2 Fig. Characteristics of the 189 participants with sufficient LM tissue for measurement of Vv(Int/cortex) at both kidney biopsies were virtually identical with those in participants without this measurement. However, the 156 participants with baseline GlomV measurements had lower AER and iGFR than those without GlomV measurements, albeit both subsets were within the normal range. At 5 years, iGFR was available for 239 participants and the mean change in iGFR over the study in this group was -7.2 mL/min/1.73 m^2^ (-1.4 ml/min/1.73 m^2^ per year). Two participants had a 5-year iGFR <60 mL/min/1.73 m^2^ (a loss of >50% of baseline iGFR).

**Table 1:** Clinical characteristics at baseline of the 243 participants in RASS with type 1 diabetes who had two kidney biopsies and measurements of plasma bradykinin and related peptides, and the subsets with cortical interstitial fractional volume and glomerular volume measurements.

Variable	Entire cohort (N = 243)	Vv(Int/cortex) subsets		*P*	GlomV subsets		*P*
		Cohort with Vv(Int/cortex) measurements (N = 189)	Cohort without Vv(Int/cortex) measurements (N = 54)		Cohort with GlomV measurements (N = 156)	Cohort without GlomV measurements (N = 87)	
RASS treatment assignment (%)							
Placebo	81 (33)	66 (35)	15 (28)	0.322	52 (33)	29 (33)	0.973
Enalapril	79 (33)	63 (33)	16 (30)		50 (32)	29 (33)	
Losartan	83 (34)	60 (32)	23 (43)		54 (35)	29 (33)	
Age (years)	29.8±9.6	30.3±9.5	28.1±9.9	0.140	29.5±9.8	30.5±9.3	0.426
Number men (%)	113 (47)	90 (48)	23 (43)	0.514	70 (45)	43 (49)	0.495
Number white (%)	237 (98)	184 (97)	53 (98)	0.740	153 (98)	84 (97)	0.463
Diabetes duration (years)	11.3±4.9	11.5±4.8	10.6±5.1	0.250	11.1±4.7	11.6±5.1	0.446
HbA1c (%)	8.5±1.6	8.5±1.5	8.7±1.8	0.268	8.6±1.5	8.5±1.6	0.663
(mmol/mol)	69.9±17.0	69.2±16.2	72.1±19.4		70.2±16.6	69.2±17.7	
Systolic BP (mm Hg)	120±12	120±12	119±12	0.682	120±12	119±11	0.813
Diastolic BP (mm Hg)	70±8	71±8	68±7	0.084	70±9	70±7	0.910
AER (μg/min)	5.0 (3.3–7.8)	5.1 (3.6–7.8)	4.8 (3.1–7.8)	0.524	4.7 (3.2–6.9)	5.9 (3.8–9.1)	0.014
iGFR (ml/min/1.73m^2^)	128±19	128±19	129±18	0.545	126±19	131±17	0.047
BK (nM)	15.0 (7.4–37.8)	15.3 (8.2–44.2)	12.3 (6.1–26.3)	0.117	13.9 (8.2–34.7)	22.2 (6.2–54.8)	0.602
BK(1–7) (nM)	38.0 (18.5–66.6)	38.4 (18.3–69.7)	35.1 (21.9–50.4)	0.350	36.1 (18.4–68.5)	41.3 (21.7–64.7)	0.726
BK(1–8) (nM)	11.7 (4.8–33.2)	11.8 (4.8–31.8)	9.6 (5.2–33.2)	0.419	11.2 (4.9–30.6)	13.7 (4.5–38.5)	0.905
Hyp3-BK (nM)	18.7 (10.3–48.7)	20.4 (10.8–50.0)	15.6 (7.3–35.0)	0.138	17.5 (10.7–46.1)	20.9 (8.4–52.5)	0.982
Hyp3-BK(1–7) (nM)	52.0 (27.7–89.7)	52.4 (29.0–88.9)	49.9 (26.4–94.8)	0.714	52.2 (29.2–87.5)	50.2 (25.7–92.0)	0.594
Hyp3-BK(1–8) (nM)	18.2 (8.9–39.7)	18.2 (9.6–41.7)	17.7 (6.5–35.3)	0.527	18.0 (9.6–38.9)	19.4 (7.7–41.7)	0.808
Unmodified peptides (nM)	64.6 (39.1–141.5)	64.5 (39.1–150.0)	66.5 (40.1–100.1)	0.516	63.7 (37.8–138.7)	75.9 (41.9–143.7)	0.373
Hydroxylated peptides (nM)	102.3 (59.8–190.9)	98.8 (59.8–199.5)	105.5 (61.6–142.0)	0.595	96.4 (60.6–177.8)	108.9 (59.8–224.9)	0.884
All peptides (nM)	167.0 (104.7–333.5)	167.0 (104.4–387.4)	167.1 (128.6–277.3)	0.747	162.6 (103.9–331.1)	172.2 (120.3–369.5)	0.550

Data are numbers (percents), means±SD, or medians (IQR). *P*-values are for differences in clinical characteristics between participants with specific measurement and those without. Abbreviations used: AER, albumin excretion rate; BP, blood pressure; HbA1c, glycosylated hemoglobin; iGFR, iohexol glomerular filtration rate; RASS, Renin-Angiotensin System Study; Vv(Int/cortex), interstitial cortical fractional volume; GlomV, mean glomerular volume BK, bradykinin; BK(1–7), bradykinin (1–7); BK(1–8), bradykinin (1–8); hyp3-BK, hydroxylated bradykinin; hyp3-BK(1–7) hydroxylated bradykinin (1–7); hyp3-BK(1–8) hydroxylated bradykinin (1–8).

Median BK(1–7) and hyp3-BK(1–7) concentrations were higher in women than men (BK(1–7) = 46 nM [IQR = 21–95] in women; 32 nM [18–50] in men, *P* = 0.001; hyp3-BK(1–7) = 70 nM [30–103] in women; 44 nM [27–75] in men, *P* = 0.005). Peptide concentrations did not differ by treatment assignment.

Baseline and 5-year follow-up morphometric characteristics are shown in Table 2. No statistically significant increase in GBM width was observed during follow-up, whereas Vv(Mes/glom) increased by 10%, Sv(PGBM/glom) decreased by 15%, and Vv(Int/cortex) increased by >50%. All these changes are in keeping with a modest increase in diabetic glomerular lesions and a moderate increase in interstitial fractional volume over the course of the study.

**Table 2:** Renal structural characteristics of the 243 participants in RASS with type 1 diabetes who had plasma bradykinin and related peptides measured at baseline.

Variable	Baseline	5 Years	*P*-value
GBM width (nm)	477±95	487±97	0.061
Vv(Mes/glom) (%)	19±4	21±5	<0.001
Vv(Int/cortex) (%)*	11±4	17±5	<0.001
Sv(PGBM/glom) (μm^2^/μm^3^)	13±2	11±2	<0.001
GlomV (x10^6^μm^3^)†	1.7±0.5	-	-
TFS/glom (x10^5^ μm^2^)†	2.2±0.7	-	-

* N = 189

^†^ N = 156

Data are means±SD. *P*-values are for changes in morphometric measurements over 5 years. Abbreviations used: GBM, glomerular basement membrane; Sv(PGBM/glom), surface density of the peripheral glomerular basement membrane; Vv(Int/cortex), interstitial cortical fractional volume; Vv(Mes/glom), mesangial fractional volume per glomerulus; GlomV, mean glomerular volume; TFS/glom, total filtration surface per glomerulus.

### BK and related peptide associations with baseline morphometric measures

None of the bradykinin related peptides correlated with baseline or change in iGFR or AER. Baseline BK(1–7) and hyp3-BK(1–7) correlated negatively with Vv(Int/cortex) and GlomV. Hyp3-BK(1–7) also correlated negatively with TFS/glom. Prior to adjustment, none of the peptides correlated with changes in any morphometric structural measures (S1 Table). Associations of BK and related peptides with GFR, AER, and morphometric measures after adjustment for standard clinical covariates (age, sex, diabetes duration, HbA1c, and MAP) and treatment assignment are shown in Table 3. BK(1–7) was weakly associated with baseline GFR. BK(1–7) and hyp3-BK(1–7) were negatively associated with Vv(Int/cortex). BK, BK(1–8), and hyp3-BK(1–8) were positively associated with TFS/glom, and BK(1–8) and hyp3-BK(1–8) were positively associated with GlomV. Hyp3-BK(1–7) was inversely associated with GlomV (S3 Fig).

**Table 3:** Parameter estimates from multivariate regression models* for the association between baseline plasma bradykinin and related peptides and the standardized baseline and log(5-year)-log baseline (Δ) morphometric variables in RASS.

Variable	BK		BK(1–7)		BK(1–8)		Hyp3-BK		Hyp3-BK(1–7)		Hyp3-BK(1–8)		Unmodified peptides		Hydroxylated peptides		Total peptides	
	β	*P*-value	β	*P*-value	β	*P*-value	β	*P*-value	β	*P*-value	β	*P*-value	β	*P*-value	β	*P*-value	β	*P*-value
GBM width																		
Baseline	-0.042	0.466	-0.108	0.068	-0.043	0.459	-0.017	0.766	-0.062	0.300	-0.016	0.780	-0.097	0.099	-0.072	0.220	-0.085	0.151
5-yr Δ	0.019	0.715	-0.037	0.489	0.029	0.580	0.063	0.223	0.007	0.899	0.061	0.235	-0.006	0.904	0.037	0.485	0.021	0.698
Vv(Mes/glom)																		
Baseline	-0.028	0.642	-0.064	0.290	-0.012	0.845	-0.054	0.359	-0.102	0.095	-0.033	0.583	-0.050	0.405	-0.090	0.135	-0.072	0.236
5-yr Δ	-0.006	0.909	-0.081	0.123	-0.000	0.993	-0.008	0.876	-0.092	0.083	-0.002	0.964	-0.048	0.354	-0.065	0.211	-0.061	0.245
Vv(Int/cortex)^†^																		
Baseline	-0.085	0.245	**-0.189**	**0.011**	-0.018	0.806	-0.082	0.257	**-0.164**	**0.027**	-0.009	0.904	-0.113	0.117	-0.110	0.129	-0.118	0.104
5-yr Δ	0.068	0.208	0.039	0.484	0.034	0.533	0.067	0.214	0.007	0.898	0.027	0.625	0.061	0.255	0.051	0.346	0.069	0.198
Sv(PGBM/glom)																		
Baseline	0.089	0.154	0.097	0.127	0.043	0.490	0.095	0.123	0.077	0.230	0.036	0.562	0.100	0.115	0.096	0.129	0.103	0.103
5-yr Δ	**0.119**	**0.013**	0.053	0.278	0.087	0.069	0.092	0.053	-0.020	0.687	0.061	0.200	**0.104**	**0.032**	0.058	0.232	0.089	0.067
GlomV‡																		
Baseline	0.113	0.169	-0.061	0.446	**0.193**	**0.019**	0.046	0.586	**-0.185**	**0.025**	**0.175**	**0.040**	0.052	0.514	-0.043	0.601	0.010	0.905
TFS/glom‡																		
Baseline	**0.166**	**0.040**	0.016	0.834	**0.211**	**0.010**	0.117	0.164	-0.102	0.209	**0.192**	**0.022**	0.112	0.153	0.030	0.710	0.077	0.330
AER																		
Baseline	-0.012	0.796	0.003	0.956	0.011	0.807	-0.012	0.792	0.021	0.659	0.022	0.631	0.016	0.737	0.033	0.472	0.016	0.730
5-yr Δ	0.083	0.109	0.071	0.177	0.076	0.141	0.086	0.090	0.071	0.181	0.079	0.122	0.083	0.113	0.093	0.074	0.096	0.065
GFR																		
Baseline	-0.002	0.851	**0.019**	**0.040**	-0.007	0.465	-0.013	0.140	0.006	0.500	-0.015	0.084	0.007	0.437	-0.005	0.612	0.003	0.733
5-yr Δ	0.056	0.063	0.108	0.092	0.048	0.439	0.071	0.252	0.103	0.106	0.058	0.356	0.064	0.314	0.073	0.245	0.071	0.262

* Adjusted for age, sex, duration of diabetes, HbA1c, MAP, and treatment assignment. Longitudinal models are also adjusted for baseline measure.

^†^ N = 189

^‡^ N = 156

Parameter estimates with *P*-values <0.05 are shown in bold. Abbreviations used: GBM, glomerular basement membrane; HbA1c, glycosylated hemoglobin; MAP, mean arterial pressure; Sv(PGBM/glom), surface density of the peripheral glomerular basement membrane; Vv(Int/cortex), interstitial cortical fractional volume; Vv(Mes/glom), mesangial fractional volume per glomerulus; GlomV, glomerular volume; TFS/glom, total filtration surface per glomerulus; BK, bradykinin; BK(1–7), bradykinin (1–7); BK(1–8), bradykinin (1–8); hyp3-BK, hydroxylated bradykinin; hyp3-BK(1–7) hydroxylated bradykinin (1–7); hyp3-BK(1–8) hydroxylated bradykinin (1–8).

**Fig 1:**
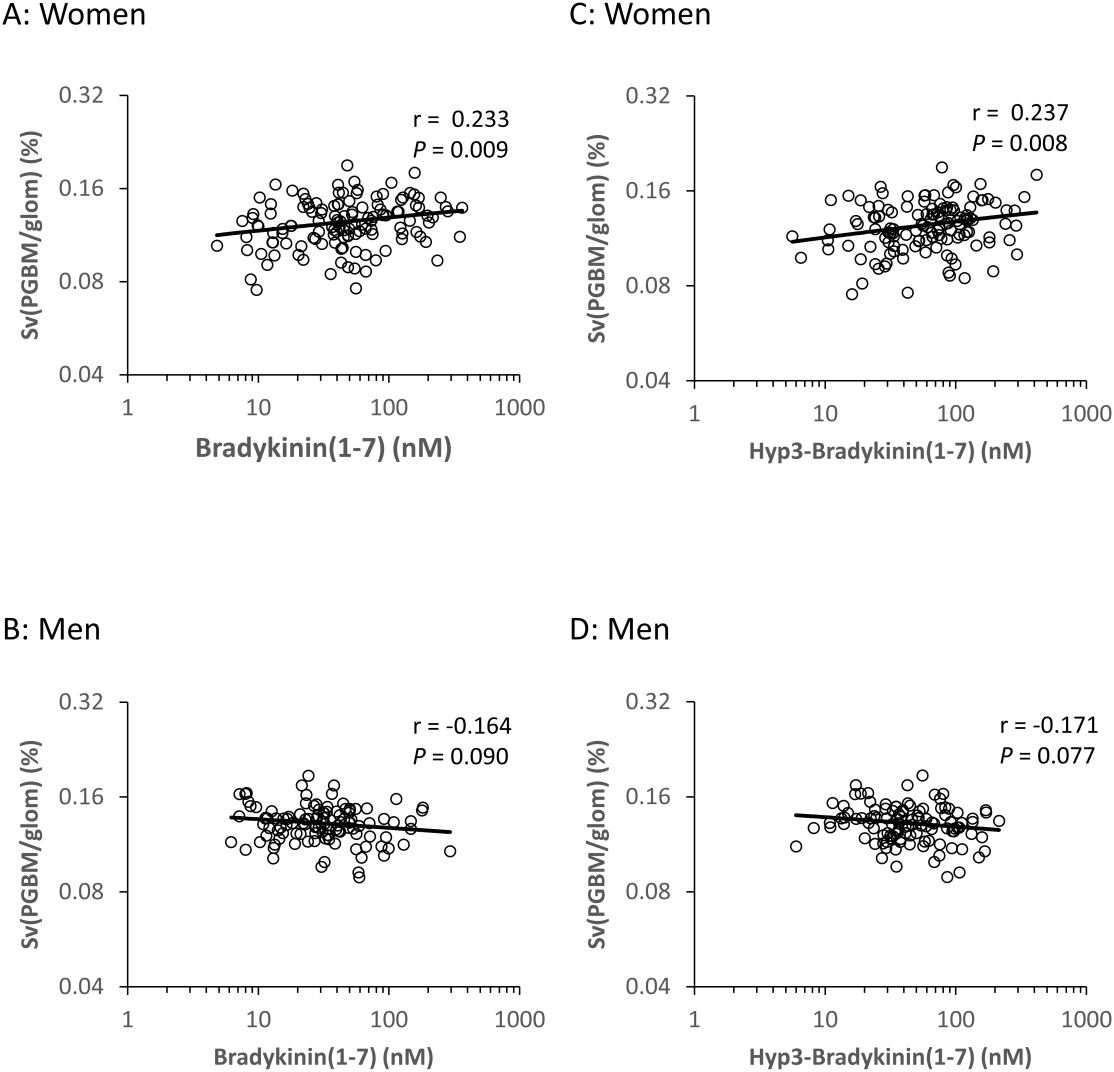
Associations of baseline plasma BK(1–7) and hyp3-BK(1–7) with baseline Sv(PGBM/glom) in the 130 women and 116 men from RASS. Residuals were computed by regressing each of the variables on baseline age, duration of diabetes, HbA1c, mean arterial pressure, and treatment assignment. Residuals are plotted on logarithmic scales.

Because of statistically significant interactions between BK(1–7) and hyp3-BK(1–7) and sex for the baseline morphometric variable Sv(PGBM/glom), we examined the relationships between these peptides and Sv(PGBM/glom) separately in men and women. BK(1–7) and hyp3-BK(1–7) were positively associated with baseline Sv(PGBM/glom) in women but not in men, suggesting that these inactive forms of BK may be renoprotective, but only in women. (Fig 1).

### BK and related peptide associations with change in morphometric measures

**Fig 2:**
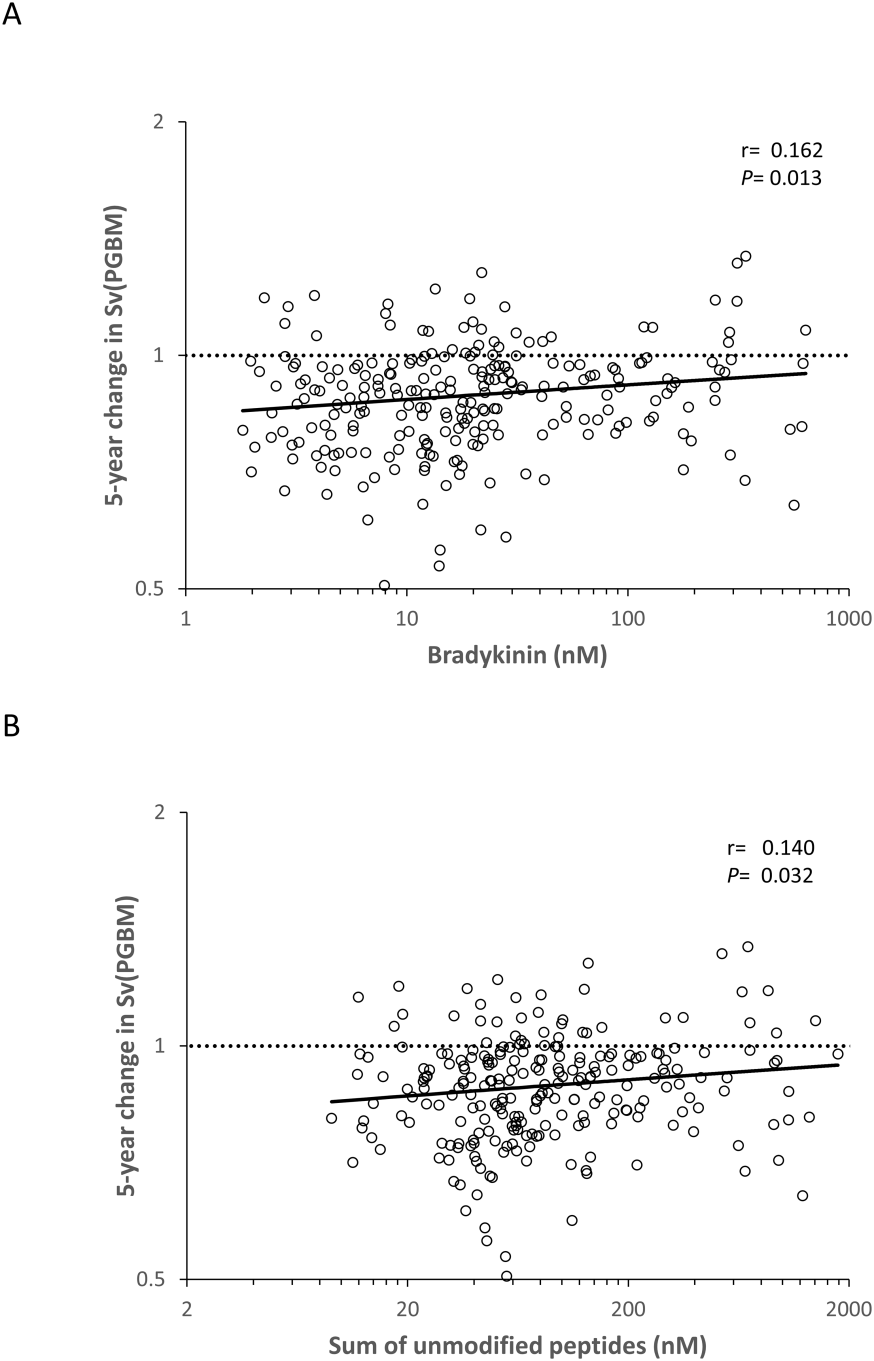
Associations of baseline plasma BK and the sum of all unmodified bradykinin peptides with the ratio (5-year/baseline) of Sv(PGBM/glom) in the 243 RASS participants. Residuals were computed by regressing each of the variables on sex, baseline age, duration of diabetes, HbA1c, mean arterial pressure, treatment assignment, and baseline Sv(PGBM/glom). Residuals are plotted on logarithmic scales. Values above the dashed line reflect an increase in Sv(PGBM/glom) and those below the line a decrease over 5 years.

**Fig 3:**
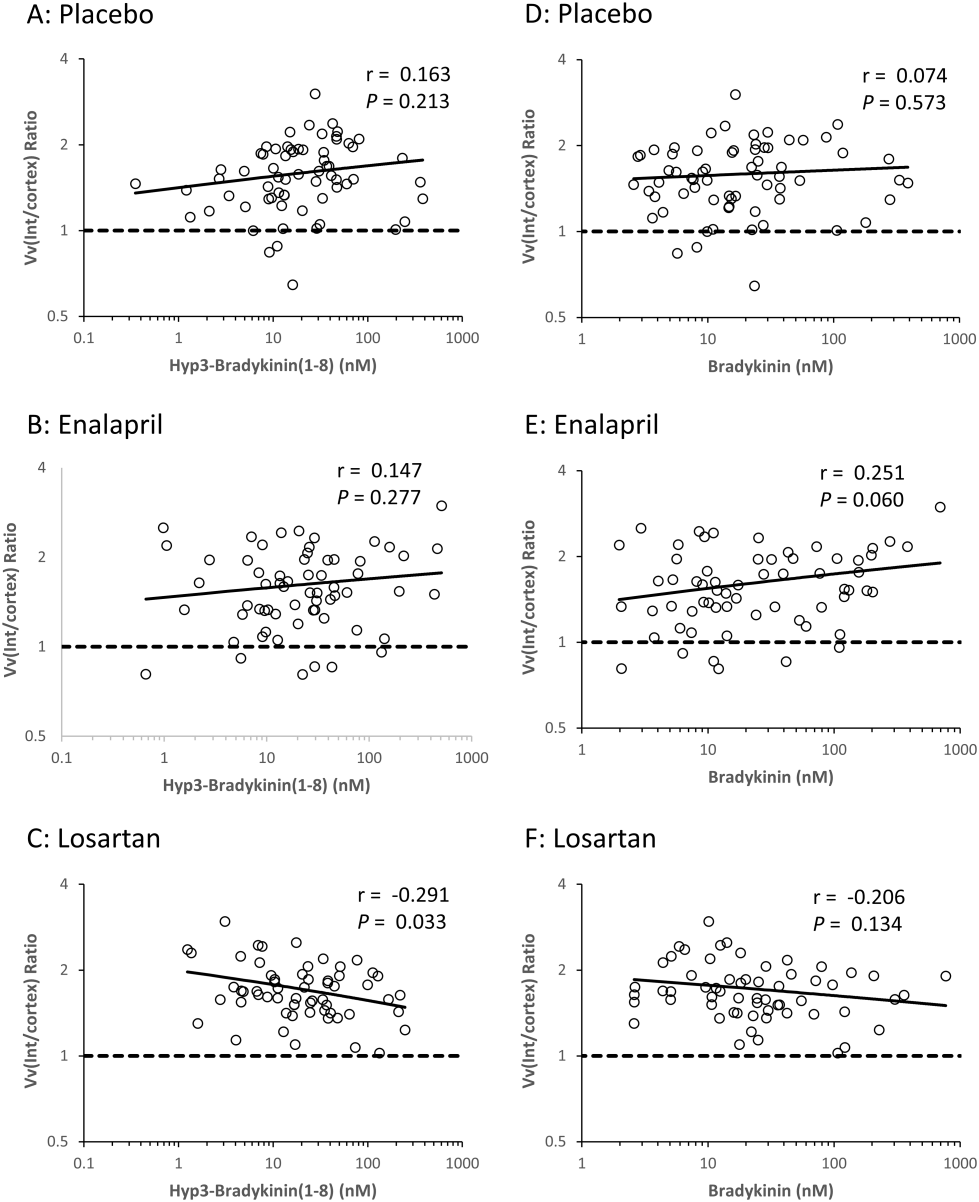
Associations of baseline BK and hyp3-BK(1–8) with change in cortical interstitial fractional volume [Vv(Int/cortex)] in the 66 participants assigned to placebo, the 63 participants assigned to enalapril, and the 60 participants assigned to losartan in RASS. Residuals were computed by regressing each of the variables on baseline age, duration of diabetes, HbA1c, mean arterial pressure, treatment assignment and baseline Vv(Int/cortex). Residuals are plotted on logarithmic scales. Values above the dashed lines reflect an increase in Vv(Int/cortex) and those below the lines a decrease over 5 years.

After further adjustment for baseline morphometric variables, BK and the sum of unmodified peptides were positively associated with change in Sv(PGBM/glom) (Fig 2). Because of statistically significant interactions between BK, hyp3-BK, and hyp3-BK(1–8) and treatment assignment for the change in Vv(Int/cortex), we examined the relationship between these peptides and change in Vv(Int/cortex) separately in the losartan, enalapril, and placebo treated groups. BK, hyp3-BK, and hyp3-BK(1–8) were negatively associated with the change in Vv(Int/cortex) in the losartan treated group but not in the placebo or enalapril treated groups (Fig 3 and S4 Fig). Although the interaction terms were statistically significant, the associations with Vv(Int/cortex) within the individual treatment assignments were mostly not. The only sub-group with a statistically significant association with Vv(Int/cortex) was the losartan treated group, and the association was with hyp3-BK(1–8) (*P* = 0.033). However, the pattern of positive associations within the enalapril treated group and negative associations within the losartan treated group were present for all three peptides.

The longitudinal findings were unchanged when we replaced the change in each morphometric variable with the 5-year measurement value alone (S2 Table). The addition of albuminuria and iGFR to the linear regression models did not affect the associations previously described (S3 Table).

## Discussion

In individuals with T1D and no clinical evidence of DN we found several modest associations between higher baseline concentrations of bradykinin and related peptides and preserved kidney structures over the 5 years of RASS, suggesting that these bradykinins may have a reno-protective role in T1D before the onset of clinically detectable DN. Specifically, we found that higher plasma concentrations of BK(1–7) and hyp3-BK(1–7) were associated with lower baseline Vv(Int/cortex) and, in women, with higher baseline Sv(PGBM/glom). In longitudinal analyses, higher plasma concentrations of BK peptides were associated with preservation of Sv(PGBM/glom), and in those treated with losartan, with less expansion of Vv(Int/cortex). The sum of all unmodified BK peptides was also significantly associated with preservation of Sv(PGBM/glom), due primarily to the associations between BK and BK(1–8) and preservation of Sv(PGBM/glom). None of these associations were strong, but the findings were consistent across several peptides and morphometric measures. These renoprotective associations may represent an early adaptive response of the kidneys to the diabetic state in which the higher plasma BK concentrations found in T1D lead to activation of B2R [11,12,24], prompting release of nitric oxide, prostanoids, prostaglandins and prostacyclin, and endothelium-derived relaxing and contracting factors that are largely protective under physiologic conditions [25, 26]. In keeping with these observations, knockout of the B2R in diabetic Akita mice results in worsening of DN [27]. In addition, in cross-sectional analyses higher concentrations of BK, BK(1–8), and hyp3-BK(1–8) were associated with higher TFS/glom, and higher concentrations of BK(1–8) and hyp3-BK(1–8) were associated with larger GlomV. On the other hand, higher concentrations of hyp3-BK(1–7) were associated with smaller GlomV. Larger GlomV is associated with relative protection from DN, in that persons with T1D and mesangial fractional volume expansion and larger glomeruli took, on average, ~10 years longer to manifest overt diabetic nephropathy than those with similar mesangial fractional volume expansion and smaller glomeruli. This finding is consistent with the concept that, within larger glomeruli, more mesangial expansion is required to compromise filtration surface [28]. Perhaps higher bradykinin concentrations are related to glomerular enlargement and to greater filtration surface in persons with T1D. However, we do not have longitudinal data for GlomV to further explore the relationship between BK and GlomV, and the potential mechanisms for such structural interactions are completely unknown.

Bradykinin is cleaved from kininogens by kallikreins and acts principally via the B2R to modulate blood pressure and inflammatory processes. BK is converted by carboxypeptidases to other biologically active forms such as BK(1–8), which has higher affinity for B1R than B2R. B1R activation stimulates release of tumor necrosis factor-α and interleukin-1, promoting inflammation and fibrosis [29,30]. Blockade of B1R in a mouse model of kidney disease reduced inflammation [13]. In healthy individuals B1R expression is low, so BK(1–8) may have limited biological effects, but in diabetes, where B1R expression is increased [31], BK(1–8) may play a greater physiologic role. BK is also metabolized to biologically inactive forms such as BK(1–7) by angiotensin 1 converting enzyme. At physiological concentrations of BK, BK(1–7) is the principal metabolite of BK metabolism in the plasma [32].

The role of the enzyme prolylcarboxypeptidase (PCRP), suggests a potential mechanism for the protective effects observed here. PCRP has many actions, including converting prekallikrein to kallikrein, increasing production of BK [33], and selectively converting the proinflammatory BK(1–8) to inactive BK(1–7) [34]. PCRP is elevated in diabetes [35, 36] and increased in diabetic Zucker rats with DN [36]. We hypothesize that in early DN, any rise in BK(1–8) and associated increased activation of B1R may be limited by PCRP activity converting BK(1–8) to its inactive form BK(1–7). In a study from the Joslin Clinic of persons with type 1 diabetes and microalbuminuria, elevated plasma concentrations of kininogen, BK(1–8), and BK(1–7) predicted renal function decline [37], indicating that, at least in more advanced DN, bradykinins are not renoprotective. It is possible that with time, the protective PCRP mechanism no longer compensates for increased production of BK(1–8), leading to increased B1R activation. In keeping with this theory, in the current study the inactive forms of BK (BK(1–7) and hyp3-BK(1–7)) were more abundant than any of the active forms, whereas in the Joslin study BK(1–8) and hyp3-BK(1–8) were both present at higher concentrations than BK(1–7). Uncertainty still exists, however, around how expression of B2R and B1R interact to either enhance or diminish renoprotection. For example, the severity of DN is increased in a mouse model lacking both receptors more than in mice lacking just the B2R, suggesting that expression of B1R is not entirely detrimental [38].

Treatment with RAS blockers modified the associations of some bradykinin peptides with change in renal morphometry. RAS blockade increases plasma BK concentration regardless of agent [17], and in rats, treatment with either enalapril or losartan reduces intrarenal BK concentrations [39]. BK, Hyp3-BK and Hyp3-BK(1–8) were negatively associated with change in Vv(Int/cortex) in the losartan treated group but not in the placebo or enalapril treated groups. We found higher concentrations of plasma BK(1–7) and hyp3-BK(1–7) and greater structural preservation in women than in men. This may be due to lower ACE activity in women than men [40,41], which increases conversion of BK to BK(1–7). We reported previously that urine monocyte chemoattractant protein-1 was associated with interstitial expansion in women but not men [6], which raises the possibility that there are sex differences in the pathophysiologic mechanisms underlying diabetic nephropathy.

Strengths of this study include the standardized processing and storage of the plasma samples at -80°C until measurement, the longitudinal design, detailed characterization of the study cohort, and use of paired kidney biopsies to provide unbiased morphometric measures of early DN structural changes. Mass spectrometric quantitation of BK sub-types also avoids antibody cross-reactivity seen in immunoassays. Weaknesses may include examination of kidney structure at a stage when changes in structure and decline in GFR are relatively modest. Six BK-related peptides were measured once in baseline samples, so we were unable to examine the change in these peptides with the change in kidney structure. Moreover, there are other BK-related peptides which may also be related to kidney structure. We also rely on plasma measures of BK and related peptides, which may not mirror BK concentrations within kidney tissue. In rats, BK concentrations were more than ten-fold higher in kidneys than in plasma [42]. Kidneys are also not the only tissue producing BK and related peptides which have also been measured in heart, lung, brain, adrenals, and fat [42]. Bradykinin and related peptides are present at higher concentrations in venous versus arterial blood, suggesting some release from tissues [43], and higher in atrial tissue than in blood [44], but we lack human data on concentrations in the kidneys. This study is exploratory and the associations found are modest, but the associations between higher BK and related peptide concentrations and structure are consistent with an overall renoprotective role for the circulating kinins. We did not adjust for multiple comparisons in our analyses because many of the variables were correlated with each other, thus leading to overcorrection. Further, this was an exploratory study, and we did not want to mask potential relationships of interest.

In summary, higher plasma concentrations of bradykinin in persons with T1D before the appearance of any clinical DN findings are associated with preserved glomerular structure and may reflect an adaptive response to diabetes that is renoprotective. These findings are consistent with animal models in which the kallikrein-kinin system has a net renoprotective effect in early DN [11, 12, 24]. An inverse association appears to emerge between bradykinin and its related peptides and progression of DN in more advanced DN [37], perhaps as the activation of B1R becomes more prominent, but the timing and determinants of this putative transition are presently unknown. The modest associations of plasma bradykinin and related peptides with morphometric measures in early DN which, while statistically significant, are likely of limited clinical value. However, these findings may help us better understand underlying mechanisms in early DN.

## Supporting information

S1 Fig Flow diagram for study. (TIF)

S2 Fig Distribution of plasma bradykinin and related peptides. A: Unadjusted values for each peptide with medians and interquartile ranges shown for each peptide. B: Log transformed and standardized values with mean and standard deviation for each peptide.
(TIF)

S3 Fig Associations of baseline bradykinin and related peptides with baseline morphometric measure in the 243 RASS participants. Residuals were computed by regressing each of the variables on baseline age, duration of diabetes, HbA1c, mean arterial pressure, and treatment assignment. Residuals are plotted on logarithmic scales.
(TIF)

S4 Fig Association of hyp3-bradykinin and ratio of 5-year to baseline Vv(Int/glom) by treatment assignment. Residuals were computed by regressing each of the variables on baseline age, duration of diabetes, HbA1c, mean arterial pressure, and treatment assignment. Residuals are plotted on logarithmic scales. *P*_interaction_ = 0.02 A: Placebo arm (N = 66). B: Enalapril arm (N = 63). C: Losartan arm: (N = 60).
(TIF)

S1 Table Pearson correlations and *P*-values between bradykinin and related peptides, functional parameters, baseline morphometric variables and changes in morphometric variables in the 243 participants with type 1 diabetes mellitus from RASS. (DOCX)

S2 Table Parameter estimates from multivariate regression models* for the association between baseline plasma bradykinin and related peptides and the standardized 5-year morphometric variables in RASS. (DOCX)

S3 Table Parameter estimates from multivariate regression models* for the association between baseline plasma bradykinin and related peptides and the standardized baseline and log(5-year)-log baseline (Δ) morphometric variables in RASS. (DOCX)

S1 File Manuscript dataset. CSV file including biomarker and morphometry data for this study.
(CSV)

S2 File Manuscript dataset metadata. Metadata for S1 File.
(TXT)
