# Supplementary figures and images for: Plasma bradykinin and early diabetic nephropathy lesions in type 1 diabetes mellitus
Source: PLoS One. 2017 Jul 10;12(7):e0180964. doi: 10.1371/journal.pone.0180964 (PMC5507314; doi:10.1371/journal.pone.0180964)

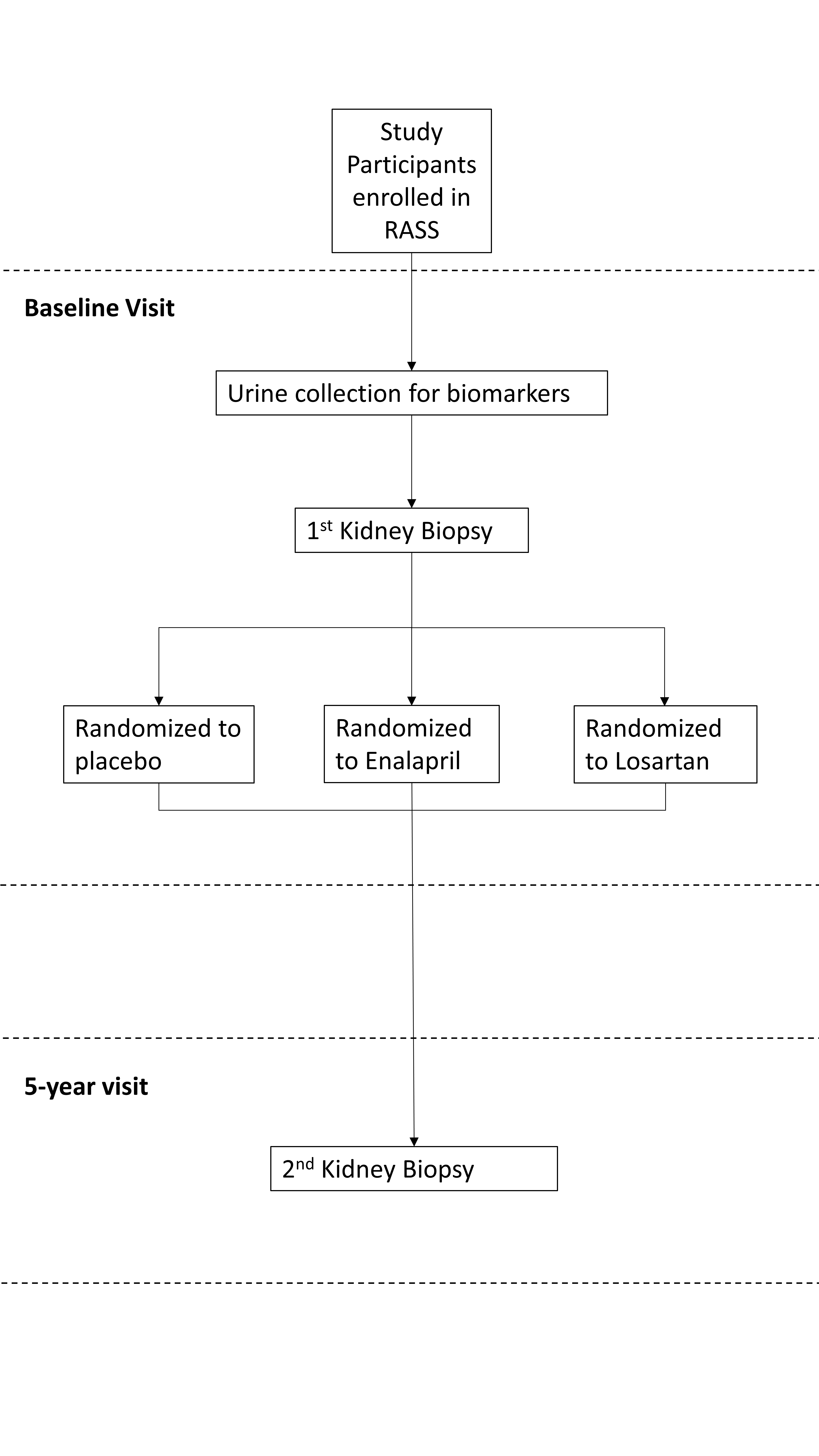

Supplement: S1 Fig — (TIF) [file pone.0180964.s001.TIF]

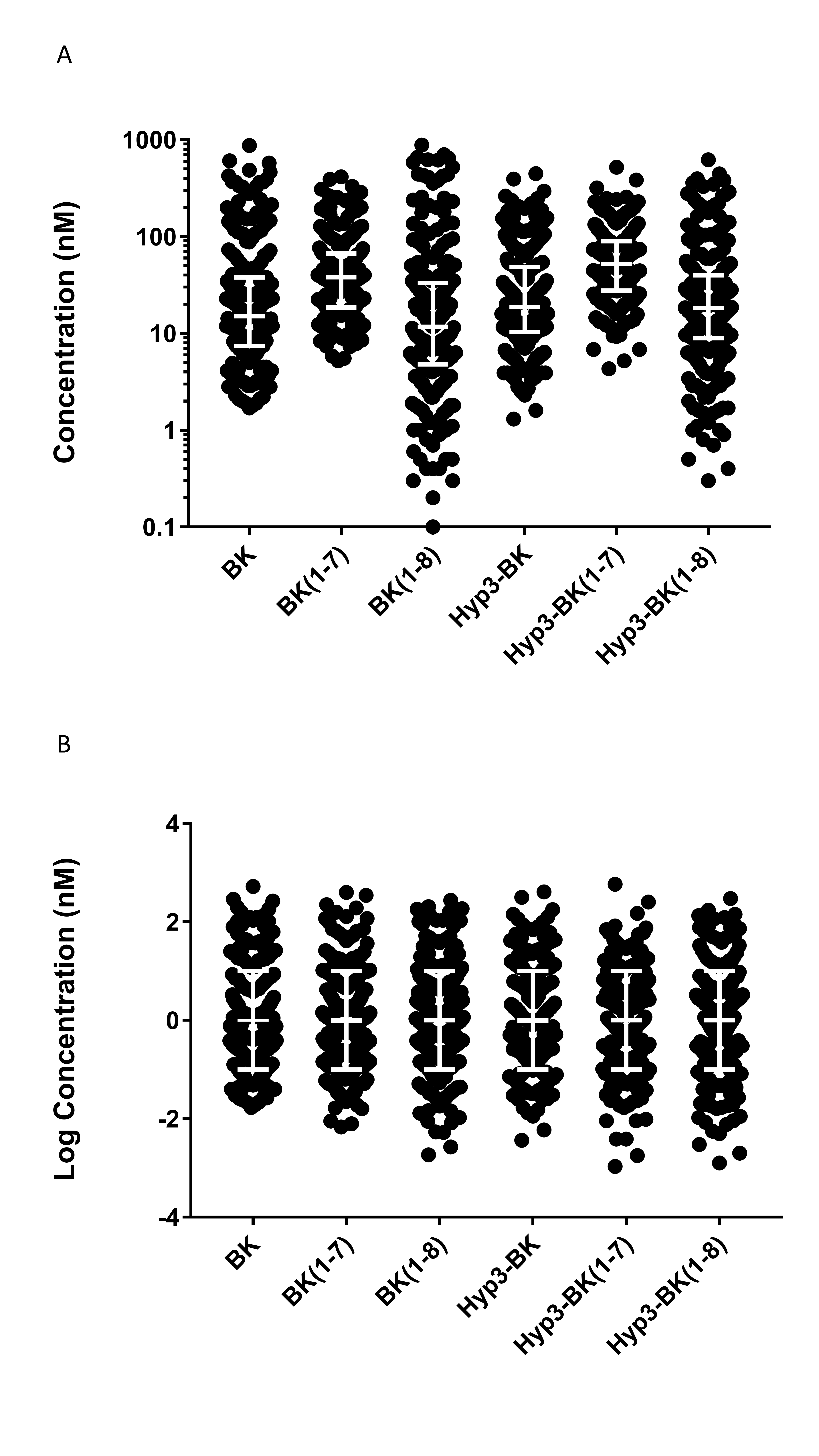

Supplement: S2 Fig — A: Unadjusted values for each peptide with medians and interquartile ranges shown for each peptide. B: Log transformed and standardized values with mean and standard deviation for each peptide. (TIF) [file pone.0180964.s002.TIF]

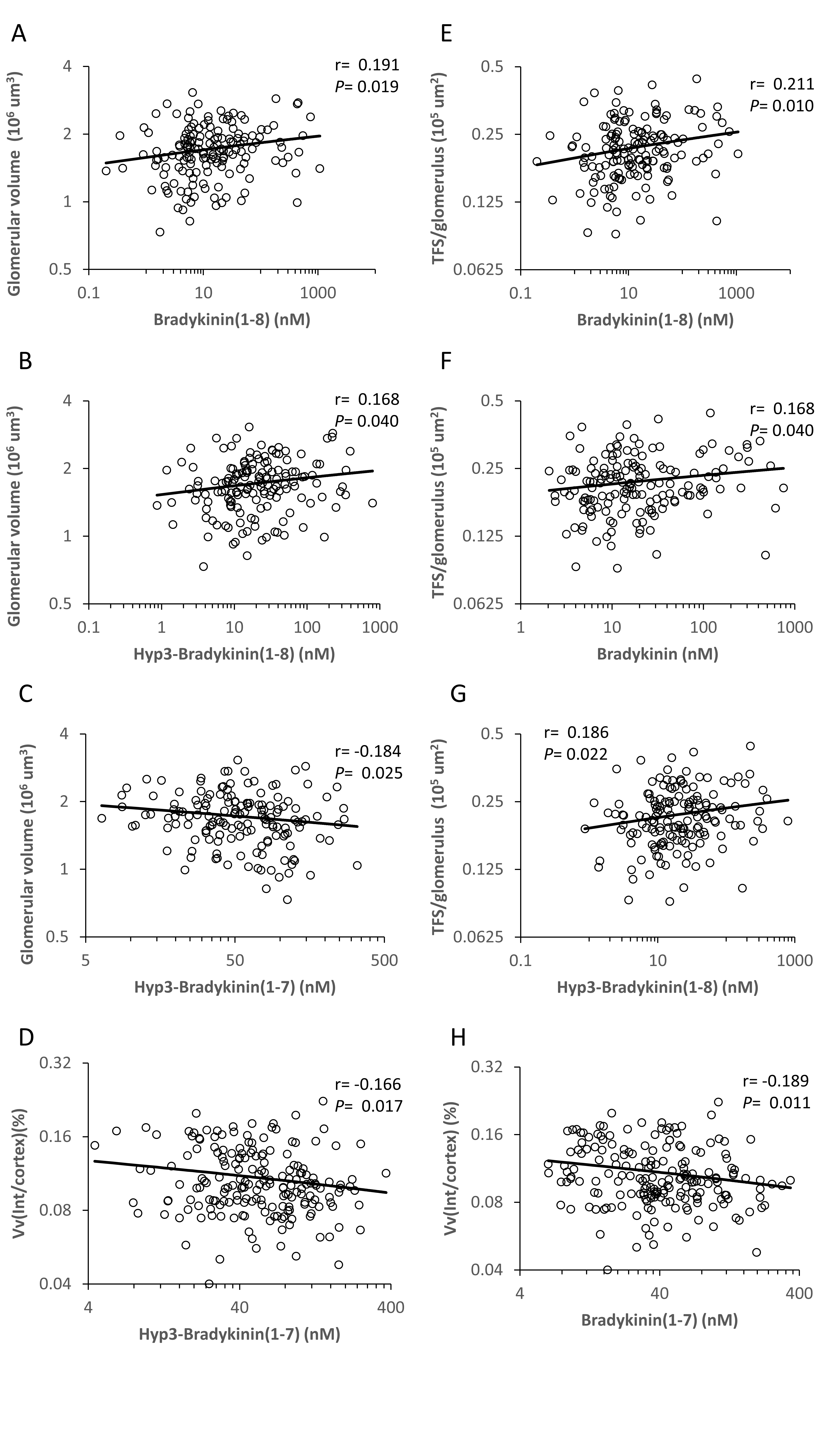

Supplement: S3 Fig — Residuals were computed by regressing each of the variables on baseline age, duration of diabetes, HbA1c, mean arterial pressure, and treatment assignment. Residuals are plotted on logarithmic scales. (TIF) [file pone.0180964.s003.TIF]

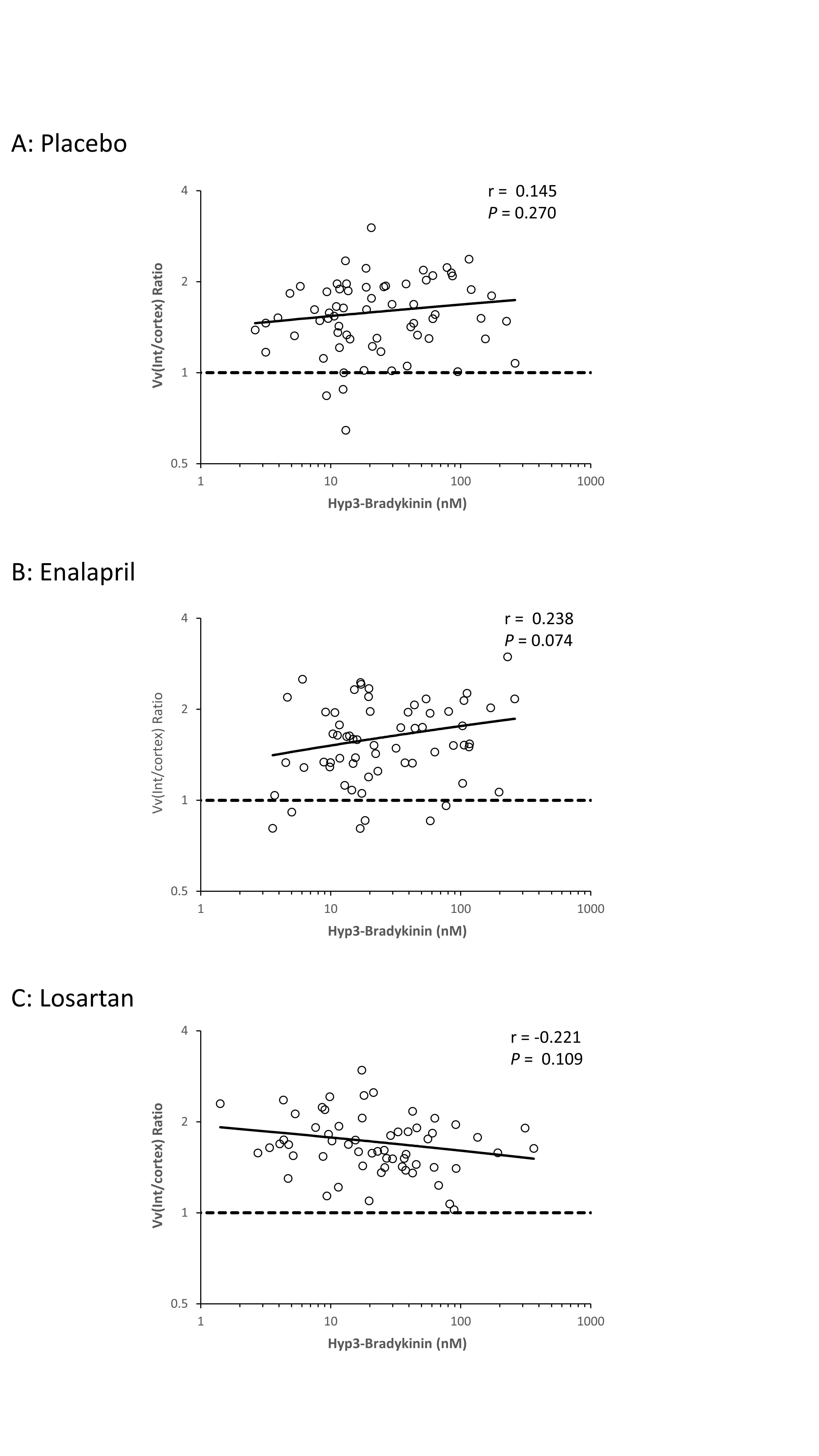

Supplement: S4 Fig — Residuals were computed by regressing each of the variables on baseline age, duration of diabetes, HbA1c, mean arterial pressure, and treatment assignment. Residuals are plotted on logarithmic scales. Pinteraction = 0.02 A: Placebo arm (N = 66). B: Enalapril arm (N = 63). C: Losartan arm: (N = 60). (TIF) [file pone.0180964.s004.TIF]
