# Supplementary material for: Plasma bradykinin and early diabetic nephropathy lesions in type 1 diabetes mellitus
Source: PLoS One. 2017 Jul 10;12(7):e0180964. doi: 10.1371/journal.pone.0180964 (PMC5507314; doi:10.1371/journal.pone.0180964)
Supplement: S1 Table — (DOCX) [file pone.0180964.s005.docx]

**S1 Table. Pearson correlations and *P*-values between bradykinin and related peptides, functional parameters, baseline morphometric variables and changes in morphometric variables in the 243 participants with type 1 diabetes mellitus from RASS.**

|  | BK | | BK(1-7) | | BK(1-8) | | Hyp3-BK | | Hyp3-BK(1-7) | | Hyp3-BK(1-8) | |
| --- | --- | --- | --- | --- | --- | --- | --- | --- | --- | --- | --- | --- |
| Clinical Variables | r | *P*-value | r | *P*-value | r | *P*-value | r | *P*-value | r | *P*-value | r | *P*-value |
| Age | 0.003 | 0.960 | **-0.162** | **0.012** | -0.020 | 0.761 | -0.033 | 0.604 | **-0.246** | **<0.001** | -0.052 | 0.420 |
| Sex | **-0.126** | **0.050** | **-0.215** | **<0.001** | -0.091 | 0.157 | -0.079 | 0.220 | **-0.184** | **0.004** | -0.064 | 0.323 |
| Diabetes duration | -0.080 | 0.211 | -0.088 | 0.172 | -0.079 | 0.222 | -0.062 | 0.334 | -0.038 | 0.556 | -0.059 | 0.359 |
| HbA1c | -0.015 | 0.811 | 0.075 | 0.244 | -0.030 | 0.641 | 0.012 | 0.847 | **0.137** | **0.033** | -0.018 | 0.782 |
| MAP | 0.075 | 0.245 | -0.052 | 0.420 | 0.100 | 0.121 | 0.036 | 0.573 | **-0.141** | **0.028** | 0.079 | 0.218 |
| iGFR | -0.030 | 0.640 | 0.110 | 0.088 | -0.046 | 0.473 | -0.079 | 0.220 | 0.063 | 0.330 | -0.087 | 0.175 |
| AER | -0.027 | 0.672 | 0.005 | 0.943 | 0.005 | 0.941 | -0.017 | 0.798 | 0.046 | 0.477 | 0.026 | 0.685 |
| Morphometric Variables | r | *P*-value | r | *P*-value | r | *P*-value | r | *P*-value | r | *P*-value | r | *P*-value |
| GBM width | -0.088 | 0.174 | -0.098 | 0.127 | -0.075 | 0.247 | -0.034 | 0.603 | 0.000 | 0.999 | -0.027 | 0.672 |
| ΔGBM width | 0.060 | 0.353 | 0.060 | 0.360 | 0.071 | 0.267 | 0.092 | 0.154 | 0.072 | 0.260 | 0.088 | 0.172 |
| Vv(Mes/glom) | -0.046 | 0.471 | -0.086 | 0.183 | -0.041 | 0.521 | -0.069 | 0.283 | -0.105 | 0.103 | -0.059 | 0.357 |
| ΔVv(Mes/glom) | 0.015 | 0.819 | -0.002 | 0.976 | 0.019 | 0.770 | 0.034 | 0.594 | 0.020 | 0.751 | 0.033 | 0.612 |
| Vv(Int/cortex)* | -0.088 | 0.227 | **-0.228** | **0.002** | -0.021 | 0.772 | -0.088 | 0.226 | **-0.216** | **0.003** | -0.012 | 0.872 |
| ΔVv(Int/cortex)* | 0.103 | 0.159 | 0.159 | 0.029 | 0.029 | 0.693 | 0.109 | 0.134 | 0.130 | 0.075 | 0.020 | 0.787 |
| Sv(PGBM/glom) | 0.080 | 0.213 | 0.088 | 0.171 | 0.044 | 0.493 | 0.099 | 0.125 | 0.074 | 0.251 | 0.043 | 0.503 |
| ΔSv(PGBM/glom) | 0.065 | 0.313 | -0.029 | 0.652 | 0.062 | 0.336 | 0.023 | 0.722 | -0.101 | 0.116 | 0.036 | 0.574 |
| GlomV† | 0.048 | 0.553 | -0.150 | 0.061 | 0.124 | 0.123 | -0.002 | 0.982 | **-0.248** | **0.002** | 0.114 | 0.156 |
| TFS/glom† | 0.083 | 0.305 | -0.074 | 0.359 | 0.127 | 0.113 | 0.047 | 0.559 | **-0.166** | **0.038** | 0.115 | 0.151 |
| Kinin Variables | r | *P*-value | r | *P*-value | r | *P*-value | r | *P*-value | r | *P*-value | r | *P*-value |
| BK | - | - | **0.688** | **<0.001** | **0.911** | **<0.001** | **0.885** | **<0.001** | **0.307** | **<0.001** | **0.819** | **<0.001** |
| BK(1-7) |  |  | - | - | **0.550** | **<0.001** | **0.574** | **<0.001** | **0.725** | **<0.001** | **0.440** | **<0.001** |
| BK(1-8) |  |  |  |  | - | - | **0.774** | **<0.001** | **0.153** | **0.017** | **0.930** | **<0.001** |
| Hyp3-BK |  |  |  |  |  |  | - | - | **0.520** | **<0.001** | **0.852** | **<0.001** |
| Hyp3-BK(1-7) |  |  |  |  |  |  |  |  | - | - | **0.295** | **<0.001** |

* N=189 † N=156

Correlations with *P*-values <0.05 are shown in bold.

Abbreviations used: HbA1c, glycosylated hemoglobin A1c; MAP, mean arterial pressure; iGFR, iohexol glomerular filtration rate; AER, albumin excretion rate; GBM, glomerular basement membrane; Sv(PGBM/glom), surface density of the peripheral glomerular basement membrane; Vv(Int/cortex), interstitial cortical fractional volume; Vv(Mes/glom), mesangial fractional volume per glomerulus; BK, bradykinin; BK(1-7), bradykinin (1-7); BK(1-8), bradykinin (1-8); hyp3-BK, hydroxylated bradykinin; hyp3-BK(1-7) hydroxylated bradykinin (1-7); hyp3-BK(1-8) hydroxylated bradykinin (1-8).
