# Supplementary material for: Plasma bradykinin and early diabetic nephropathy lesions in type 1 diabetes mellitus
Source: PLoS One. 2017 Jul 10;12(7):e0180964. doi: 10.1371/journal.pone.0180964 (PMC5507314; doi:10.1371/journal.pone.0180964)
Supplement: S2 Table — (DOCX) [file pone.0180964.s006.docx]

**S2 Table. Parameter estimates from multivariate regression models* for the association between baseline plasma bradykinin and related peptides and the standardized 5-year morphometric variables in RASS.**

| Variable | BK | | BK(1-7) | | BK(1-8) | | Hyp3-BK | | Hyp3-BK(1-7) | | Hyp3-BK(1-8) | | Unmodified peptides | | Hydroxylated peptides | | Total peptides | |
| --- | --- | --- | --- | --- | --- | --- | --- | --- | --- | --- | --- | --- | --- | --- | --- | --- | --- | --- |
|  | β | *P*-value | β | *P*-value | β | *P*-value | β | *P*-value | β | *P*-value | β | *P*-value | β | *P*-value | β | *P*-value | β | *P*-value |
| GBM width | 0.014 | 0.715 | -0.028 | 0.489 | 0.022 | 0.580 | 0.047 | 0.223 | 0.005 | 0.899 | 0.046 | 0.235 | -0.005 | 0.904 | 0.028 | 0.485 | 0.016 | 0.698 |
| Vv(Mes/glom) | -0.006 | 0.909 | -0.088 | 0.123 | -0.001 | 0.993 | -0.009 | 0.876 | -0.100 | 0.083 | -0.002 | 0.964 | -0.052 | 0.354 | -0.071 | 0.211 | -0.066 | 0.245 |
| Vv(Int/cortex)^†^ | 0.092 | 0.208 | 0.053 | 0.484 | 0.046 | 0.533 | 0.091 | 0.214 | 0.010 | 0.898 | 0.037 | 0.625 | 0.083 | 0.255 | 0.069 | 0.346 | 0.094 | 0.198 |
| Sv(PGBM/glom) | **0.151** | **0.013** | 0.067 | 0.278 | 0.110 | 0.069 | 0.116 | 0.053 | -0.025 | 0.687 | 0.077 | 0.200 | **0.132** | **0.032** | 0.074 | 0.232 | 0.113 | 0.067 |

* Adjusted for age, sex, duration of diabetes, HbA1c, MAP, treatment assignment, and baseline structure.

† N=189

Parameter estimates with *P*-values <0.05 are shown in bold. Abbreviations used: GBM, glomerular basement membrane; Sv(PGBM/glom), surface density of the peripheral glomerular basement membrane; Vv(Int/cortex), interstitial cortical fractional volume; Vv(Mes/glom), mesangial fractional volume per glomerulus; BK, bradykinin; BK(1-7), bradykinin (1-7); BK(1-8), bradykinin (1-8); hyp3-BK, hydroxylated bradykinin; hyp3-BK(1-7) hydroxylated bradykinin (1-7); hyp3-BK(1-8) hydroxylated bradykinin (1-8).
