# Supplementary material for: Plasma bradykinin and early diabetic nephropathy lesions in type 1 diabetes mellitus
Source: PLoS One. 2017 Jul 10;12(7):e0180964. doi: 10.1371/journal.pone.0180964 (PMC5507314; doi:10.1371/journal.pone.0180964)
Supplement: S3 Table — (DOCX) [file pone.0180964.s007.docx]

**S3 Table. Parameter estimates from multivariate regression models* for the association between baseline plasma bradykinin and related peptides and the standardized baseline and log(5-year)-log baseline (∆) morphometric variables in RASS.**

| Variable | BK | | BK(1-7) | | BK(1-8) | | Hyp3-BK | | Hyp3-BK(1-7) | | Hyp3-BK(1-8) | | Unmodified peptides | | Hydroxylated peptides | | Total peptides | |
| --- | --- | --- | --- | --- | --- | --- | --- | --- | --- | --- | --- | --- | --- | --- | --- | --- | --- | --- |
|  | β | *P*-value | β | *P*-value | β | *P*-value | β | *P*-value | β | *P*-value | β | *P*-value | β | *P*-value | β | *P*-value | β | *P*-value |
| GBM width |  |  |  |  |  |  |  |  |  |  |  |  |  |  |  |  |  |  |
| Baseline | -0.043 | 0.465 | -0.104 | 0.082 | -0.045 | 0.446 | -0.021 | 0.718 | -0.060 | 0.318 | -0.020 | 0.729 | -0.095 | 0.108 | -0.073 | 0.217 | -0.083 | 0.159 |
| 5-yr ∆ | 0.019 | 0.722 | -0.045 | 0.405 | 0.032 | 0.544 | 0.067 | 0.194 | 0.006 | 0.915 | 0.069 | 0.184 | -0.009 | 0.873 | 0.041 | 0.443 | 0.020 | 0.706 |
| Vv(Mes/glom) |  |  |  |  |  |  |  |  |  |  |  |  |  |  |  |  |  |  |
| Baseline | -0.026 | 0.660 | -0.075 | 0.222 | -0.011 | 0.859 | -0.048 | 0.419 | -0.108 | 0.078 | -0.029 | 0.626 | -0.056 | 0.353 | -0.093 | 0.125 | -0.076 | 0.211 |
| 5-yr ∆ | -0.005 | 0.927 | -0.076 | 0.149 | -0.004 | 0.942 | -0.011 | 0.830 | -0.093 | 0.079 | -0.010 | 0.844 | -0.048 | 0.358 | -0.071 | 0.171 | -0.062 | 0.237 |
| Vv(Int/cortex)^†^ |  |  |  |  |  |  |  |  |  |  |  |  |  |  |  |  |  |  |
| Baseline | -0.087 | 0.228 | **-0.189** | **0.011** | -0.018 | 0.806 | -0.082 | 0.258 | **-0.169** | **0.022** | -0.013 | 0.858 | -0.121 | 0.093 | -0.117 | 0.107 | -0.1231 | 0.087 |
| 5-yr ∆ | 0.069 | 0.204 | 0.040 | 0.478 | 0.036 | 0.513 | 0.069 | 0.206 | 0.008 | 0.886 | 0.030 | 0.593 | 0.063 | 0.247 | 0.053 | 0.329 | 0.071 | 0.192 |
| Sv(PGBM/glom) |  |  |  |  |  |  |  |  |  |  |  |  |  |  |  |  |  |  |
| Baseline | 0.089 | 0.155 | 0.099 | 0.122 | 0.043 | 0.495 | 0.095 | 0.125 | 0.078 | 0.231 | 0.035 | 0.571 | 0.100 | 0.115 | 0.095 | 0.132 | 0.103 | 0.104 |
| 5-yr ∆ | **0.117** | **0.013** | 0.058 | 0.235 | 0.088 | 0.062 | 0.088 | 0.062 | -0.015 | 0.756 | 0.063 | 0.184 | **0.109** | **0.024** | 0.063 | 0.188 | 0.092 | 0.054 |
| GlomV‡ |  |  |  |  |  |  |  |  |  |  |  |  |  |  |  |  |  |  |
| Baseline | 0.119 | 0.147 | -0.036 | 0.659 | **0.188** | **0.022** | 0.048 | 0.576 | **-0.165** | **0.047** | 0.165 | 0.054 | 0.064 | 0.423 | -0.035 | 0.675 | 0.022 | 0.788 |
| TFS/glom‡ |  |  |  |  |  |  |  |  |  |  |  |  |  |  |  |  |  |  |
| Baseline | **0.170** | **0.036** | 0.033 | 0.680 | **0.208** | **0.011** | 0.119 | 0.160 | -0.091 | 0.274 | **0.187** | **0.027** | 0.120 | 0.130 | 0.036 | 0.662 | 0.085 | 0.287 |

* Adjusted for age, sex, duration of diabetes, HbA1c, MAP, treatment assignment, ln(AER), and iGFR. Longitudinal models are also adjusted for baseline structure.

† N=189 ‡ N=156

Parameter estimates with *P*-values <0.05 are shown in bold. Abbreviations used: GBM, glomerular basement membrane; HbA1c, glycosylated hemoglobin; MAP, mean arterial pressure; Sv(PGBM/glom), surface density of the peripheral glomerular basement membrane; Vv(Int/cortex), interstitial cortical fractional volume; Vv(Mes/glom), mesangial fractional volume per glomerulus; GlomV, glomerular volume; TFS/glom, total filtration surface per glomerulus; BK, bradykinin; BK(1-7), bradykinin (1-7); BK(1-8), bradykinin (1-8); hyp3-BK, hydroxylated bradykinin; hyp3-BK(1-7) hydroxylated bradykinin (1-7); hyp3-BK(1-8) hydroxylated bradykinin (1-8).
